# Characterisation of Weibel–Palade body fusion by amperometry in endothelial cells reveals fusion pore dynamics and the effect of cholesterol on exocytosis

**DOI:** 10.1242/jcs.138438

**Published:** 2013-12-01

**Authors:** Emma A. Cookson, Ianina L. Conte, John Dempster, Matthew J. Hannah, Tom Carter

**Affiliations:** 1 MRC National Institute for Medical Research, Mill Hill, London, NW7 1AA, UK; 2 University of Strathclyde, Glasgow G1 1XQ, UK; 3 Microbiology Services Division Colindale, Public Health England, 61 Colindale Avenue, London, NW9 5EQ, UK

**Keywords:** Weibel–Palade bodies, Endothelial cell, VMAT1, Serotonin, Amperometry, Exocytosis

## Abstract

Regulated secretion from endothelial cells is mediated by Weibel–Palade body (WPB) exocytosis. Plasma membrane cholesterol is implicated in regulating secretory granule exocytosis and fusion pore dynamics; however, its role in modulating WPB exocytosis is not clear. To address this we combined high-resolution electrochemical analysis of WPB fusion pore dynamics, by amperometry, with high-speed optical imaging of WPB exocytosis following cholesterol depletion or supplementation in human umbilical vein endothelial cells. We identified serotonin (5-HT) immunoreactivity in WPBs, and VMAT1 expression allowing detection of secreted 5-HT as discrete current spikes during exocytosis. A high proportion of spikes (∼75%) had pre-spike foot signals, indicating that WPB fusion proceeds via an initial narrow pore. Cholesterol depletion significantly reduced pre-spike foot signal duration and increased the rate of fusion pore expansion, whereas cholesterol supplementation had broadly the reverse effect. Cholesterol depletion slowed the onset of hormone-evoked WPB exocytosis, whereas its supplementation increased the rate of WPB exocytosis and hormone-evoked proregion secretion. Our results provide the first analysis of WPB fusion pore dynamics and highlight an important role for cholesterol in the regulation of WPB exocytosis.

## Introduction

Weibel–Palade bodies (WPBs) are regulated secretory granules of endothelial cells (Knipe et al., 2010; Rondaij et al., 2006). WPB formation is driven by condensation of polymers of the haemostatic glycoprotein Von Willebrand factor (VWF) in association with the cleaved VWF propolypeptide (proregion) (Sadler, 1998; Springer, 2011; Wagner, 1990). Secreted VWF plays important roles in haemostasis, might modulate blood vessel growth and the spread of metastatic cancer cells, and contributes to thrombosis under pathological conditions (Sadler, 1998; Starke et al., 2011; Terraube et al., 2007). WPB exocytosis is also a main route for regulated secretion of proteins modulating inflammation, blood vessel tone and vessel growth and remodelling (Rondaij et al., 2006; van Breevoort et al., 2012).

Physical, mechanical and/or chemical signals generated during vessel injury, stress or infection trigger WPB exocytosis, primarily through elevating intracellular free calcium ion ([Ca^2+^]_i_) and/or cAMP concentrations (Rondaij et al., 2006). Key molecules involved in the delivery, docking and exocytosis of WPBs have been identified (Bierings et al., 2012; Fu et al., 2005; König et al., 1998; Nightingale et al., 2009; Pulido et al., 2011; Rojo Pulido et al., 2011; Zografou et al., 2012); however, little is known about the processes regulating the final stages of WPB exocytosis, in particular the dynamics of the fusion pore connecting the WPB lumen with the extracellular space. Fusion pore dynamics are modulated by Ca^2+^, proteins and membrane lipids, such as cholesterol (Anantharam et al., 2011; Berberian et al., 2009; Chang et al., 2009; Fang et al., 2008; Ge et al., 2010; Jackson and Chapman, 2008; Neco et al., 2008; Segovia et al., 2010; Wang et al., 2010; Zimmerberg and Chernomordik, 2005). Regulation of fusion pore dynamics is thought to influence both the extent and composition of secreted cargo (Braun et al., 2007; Obermüller et al., 2005; Soekmadji and Thorn, 2010); Vardjan et al., 2009; Vardjan et al., 2007), a process recently reported to occur during WPB exocytosis (Babich et al., 2008). Although high levels of circulating cholesterol are associated with elevated plasma VWF (Blann et al., 1995; Pérez-Jiménez et al., 1999), a risk factor for thrombosis and stroke (Jansson et al., 1991; Qizilbash et al., 1997), it remains unclear whether endothelial cell plasma membrane cholesterol modulates WPB fusion pore dynamics or stimulated cargo secretion.

Fusion pores form on a millisecond time scale and require specialised approaches for their detection. Where secretory granules contain diffusible oxidisable molecules, e.g. monoamines, fusion pore formation and expansion can be resolved as current spikes using carbon fibre amperometry (amperometry) (Chow and Von Ruden, 2009). The shape of an amperometric current spike can provide direct mechanistic information about the underlying process of membrane fusion, including the initial formation of the fusion pore, seen as a small increase in current preceding the main spike and termed the pre-spike foot signal (Alvarez de Toledo et al., 1993; Chow et al., 1992).

Here, we report that human umbilical vein endothelial cells (HUVECs) can contain serotonin (5-HT)-positive WPBs. We show that HUVECs express the vesicular monoamine transporter 1 (VMAT1), and that epitope-tagged VMAT1 localises to WPBs and mediates WPB 5-HT sequestration. Using this new information we applied amperometry, to measure 5-HT release from WPBs. Combining amperomerty with high-speed fluorescence imaging of WPB exocytosis in live HUVECs, we have analysed the properties of the WPB fusion pore formed during Ca^2+^-evoked exocytosis, and the modulation of fusion pore dynamics and WPB exocytosis by depletion or supplementation of cholesterol. Our data highlight an important role for plasma membrane cholesterol in the regulation of WPB exocytosis from endothelial cells.

## Results

### 5-HT is present in WPBs of some HUVECs

**Fig. 1.**
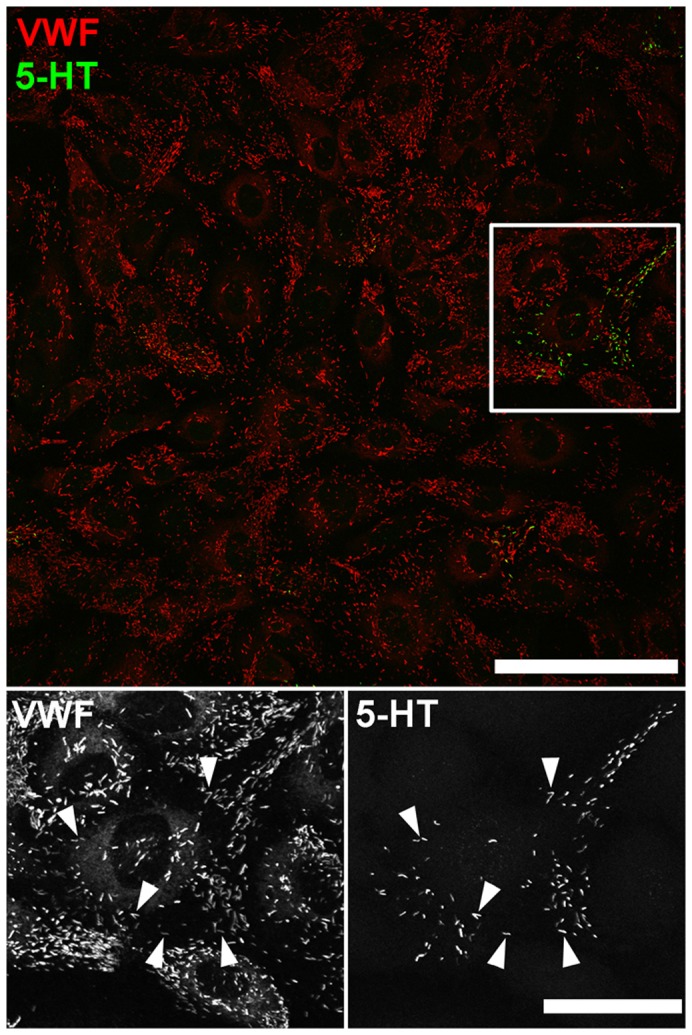
**5-HT immunoreactivity localises to WPBs in HUVECs.** Image shows a field of HUVECs immunolabeled with specific antibodies to VWF (red, rabbit Ab) and 5-HT (green). Scale bar: 100 µm. The region indicated by the white box is shown at an expanded scale in the lower panels. The proportion of cells containing WPB 5-HT immunoreactivity ranged from 0–5.2±1.0% [determined from analysis of 48 randomly selected low power (40×magnification) fields]. Scale bar: 20 µm. Immunofluorescence images of fixed cells shown here and in subsequent figures were taken at room temperature using Leica SP1 or SP2 confocal microscopes and software (Mannheim, Germany) equipped with 40×, 63× or 100×objectives (SP1; PL APO40× 1.25–0.75 NA, PL APO100× 1.4NA, SP2; HCX PL APO40× 1.2 NA, PL APO 63× 1.4NA, PL APO100× 1.4NA).

Many cells secrete monoamines, which can be detected using amperometry (Chow and Von Ruden, 2009). To examine whether WPBs might contain such molecules we first looked for evidence of endogenous immunoreactivity for the monoamines dopamine, noradrenaline, adrenaline or 5-HT in WPBs. No evidence for WPB dopamine, noradrenaline or adrenaline immunoreactivity was detected; however, we discovered 5-HT immunoreactivity in WPBs in up to 5% of cells (Fig. 1). The relatively low numbers of cells with WPB 5-HT immunoreactivity suggested that electrochemical detection of secreted 5-HT could be problematic. To test this, we applied amperometry to fura-2-loaded HUVECs expressing proregion–EGFP to fluorescently label WPBs. The carbon fibre microelectrode was positioned over regions of the cell containing multiple fluorescent WPBs. Cells were stimulated by gentle mechanical pressure of the electrode on the cell surface, which produced an increase in [Ca^2+^]_i_ and exocytosis of fluorescent WPBs, the kinetics of which are summarised and discussed in supplementary material Fig. S1A. Simultaneous amperometric recording revealed no current spikes associated with exocytosis of fluorescent WPBs under the electrode (supplementary material Fig. S1B), consistent with WPBs in most cells lacking secretable monoamines. Nonetheless, the presence of 5-HT immunoreactivity in some WPBs led us to investigate ways to load WPBs with this monoamine for amperometric analysis of WPB exocytosis.

### HUVECs express VMAT1, and EGFP–VMAT1 localises to WPBs resulting in both WPB 5-HT immunoreactivity and current spikes during exocytosis

5-HT immunoreactivity in WPBs suggested the existence of a VMAT on the organelle. RT-PCR of HUVEC cDNA revealed the presence of VMAT1 (encoded by *SLC18A1*) but not VMAT2 (encoded by *SLC18A2*) mRNA. In addition, we found mRNA for the plasma membrane monoamine transporter (PMAT, encoded by *SLC29A4*) but not the serotonin transporter (SERT, encoded by *SLC6A4*) or the organic cation transporter 3 (OCT3, encoded by *SLC22A3*) (supplementary material Fig. S1C). Immunocytochemistry was attempted to establish the subcellular localisation of endogenous VMAT1, however, we were unable to detect an endogenous signal with the reagents used. We therefore expressed epitope-tagged VMAT1 to assess the putative subcellular localisation of this VMAT in HUVECs.

**Fig. 2.**
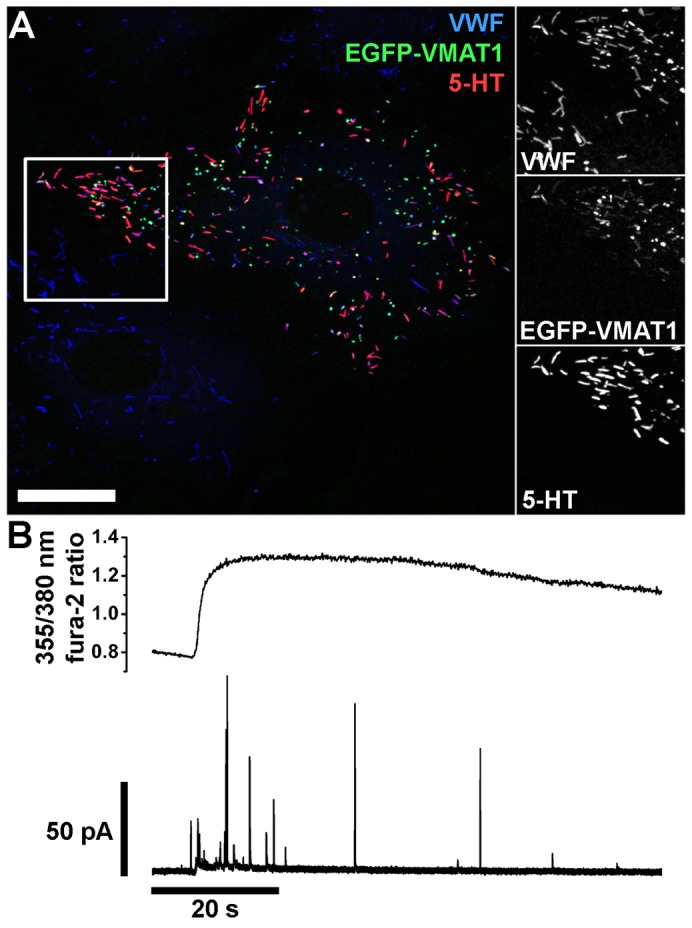
**EGFP–VMAT1 localises to WPBs, and results in the appearance of WPB 5-HT immunoreactivity and current spikes during endothelial cell stimulation.** (A) HUVECs expressing EGFP–VMAT1 and immunolabelled with specific antibodies to VWF (blue, rabbit Ab), EGFP (green) and 5-HT (red). Scale bar: 20 µm. Regions indicated by white boxes are shown in grey scale to the right at an expanded scale. (B) Simultaneous recording of fura-2 fluorescence ratio (top trace) and electrode current (bottom trace) from a single proregion–EGFP- and mCherry–VMAT1-expressing HUVECs following mechanical stimulation with the electrode.

EGFP–VMAT1 localised to WPBs, which showed striking WPB 5-HT immunoreactivity (Fig. 2A). No 5-HT immunoreactivity was detected in any other subcellular structures, and the effect was specific for EGFP–VMAT1; expressed EGFP–VAChT (vesicular acetylcholine transporter) also localised to WPBs but did not produce WPB 5-HT immunoreactivity (supplementary material Fig. S2A). Consistent with WPB VMAT1 mediating 5-HT sequestration, reserpine treatment abolished WPB 5-HT immunoreactivity in EGFP–VMAT1-expressing cells (supplementary material Fig. S2B). We measured the intra-WPB pH (pH_WPB_) in epitope-tagged VMAT1-expressing cells, to test whether VMAT1 expression perturbed the pH_WPB_, but found no differences compared with control cells (supplementary material Fig. S2C). To identify the source of 5-HT in WPBs we focused on the tissue culture fetal calf serum, which is reported to contain up to 7 µM 5-HT (Hamamori et al., 1988; Little et al., 2002; McEwan and Parsons, 1987; Mothersill et al., 2010). 5-HT was first discharged from EGFP–VMAT1-expressing WPBs by exposure to NH_4_Cl, after which the cells were incubated in full growth medium, serum-free growth medium or dialysed medium (see Materials and Methods) for 5 hours. In full growth medium WPB 5-HT immunoreactivity was restored; however, in serum-free growth medium or dialysed medium WPB 5-HT immunoreactivity was absent. WPB 5-HT immunoreactivity could be restored by brief exposure to serum-free growth medium or dialysed medium supplemented with ≥0.3 µM 5-HT. In these cases, WPB 5-HT immunoreactivity remained detectable for up to 16 hours in 5-HT- and serum-free growth medium or dialysed medium (e.g. supplementary material Fig. S3A–C).

**Fig. 3.**
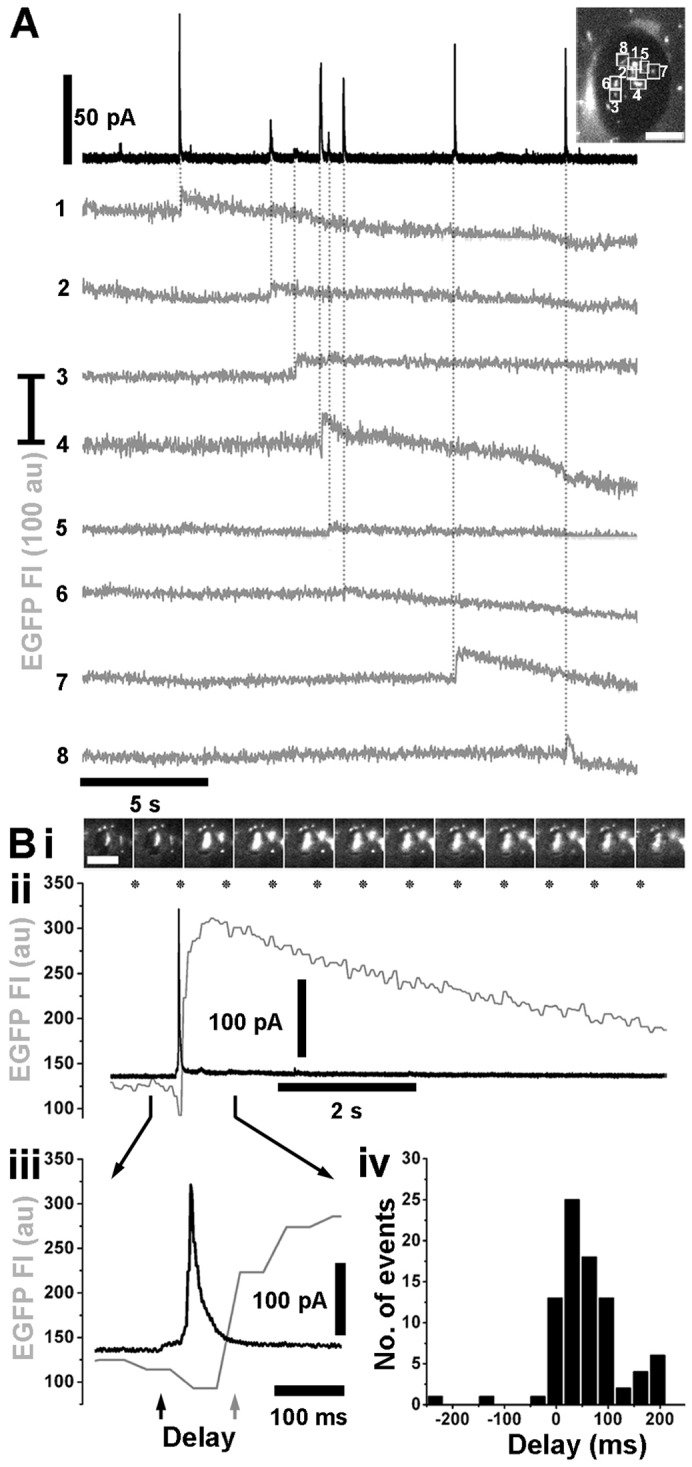
**Current spikes are associated with WPB fusion.** (A) Representative recording of electrode current (black trace) and fluorescence intensity of individual EGFP-labelled WPBs (grey traces, 1–8) in an mCherry–VMAT1 and proregion–EGFP co-expressing HUVEC. Positions of individual EGFP-labelled WPBs (numbered 1–8) beneath the electrode, just prior to stimulation, are shown in the image of the electrode footprint (top right). Scale bar: 5 µm. A current spike is associated with fusion of each WPB (marked by vertical dotted lines). (Bi) Image montage showing the fusion of a single proregion–EGFP-containing WPB isolated beneath an electrode. Images of EGFP fluorescence were acquired at 15 frames/second, and correspond to the times indicated by the asterisks in Bii. Scale bar: 5 µm. (Bii–iii) The temporal association between the increase in WPB EGFP fluorescence intensity, due to fusion (grey traces), and the onset of the current spike (black traces). There is a delay between the onset of the current spike (black arrow in Biii) and the onset of the rise in WPB EGFP fluorescence intensity (grey arrow in Biii). (Biv) A summary of the distribution of delay times (bin width, 33 ms; mean 57.4±7.27 ms, *n* = 84).

Applying amperometry to fura-2-loaded cells coexpressing mCherry–VMAT1 and proregion–mEGFP revealed clear current spikes during cell stimulation (Fig. 2B). Simultaneous optical detection of WPB exocytosis revealed that individual current spikes (Fig. 3A, black trace) were associated with individual WPB fusion events (grey traces). Close inspection revealed a delay between the onset of a current spike and the increase in WPB EGFP fluorescence intensity (Fig. 3B). Spike quantal size (*Q*) significantly correlated with WPB length (volume) indicating that the concentration of oxidisable species within each individual WPB is similar (supplementary material Fig. S3D). No current spikes were detected in the absence of stimulation, or during stimulation when recording from regions lacking fluorescent WPBs; consistent with current spikes arising solely from WPB exocytotic events.

### Properties of current spikes associated with WPB fusion

**Fig. 4.**
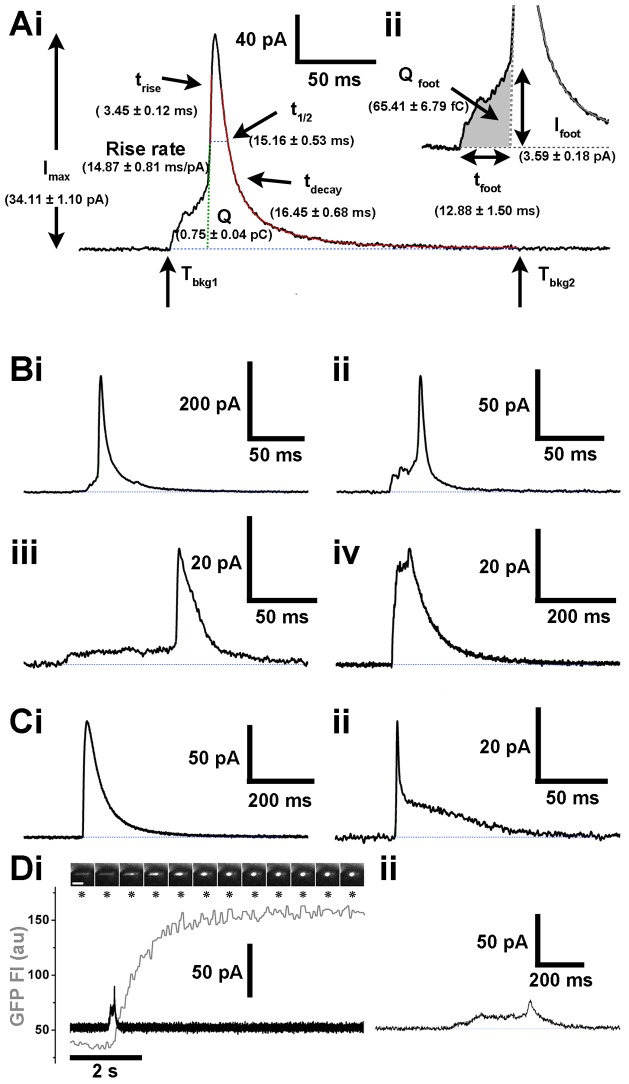
**Electrochemical characterisation of WPB exocytosis – WPBs form a restricted pore prior to full exocytosis.** (Ai) Current spike parameters were calculated between the times when current exceeded five s.d. of the baseline (*T*_bkg1_) and subsequently returned to the baseline (*T*_bkg2_) and included maximum current amplitude (*I*_max_), area (*Q*), width of the spike at half the amplitude (*t*_1/2_), duration of the rise (*t*_rise_) and decay (*t*_decay_) phases between 25% and 75% of the *I*_max_ and rate of rise (pA/ms). (Aii) Pre-spike foot signal parameters were *t*_foot_ [which corresponds to the time between *T*_bkg1_ and the pre-spike foot signal end as defined by the interception of the rise slope with the baseline (Mosharov and Sulzer)], *Q*_foot_ (shaded area) and *I*_foot_ [the average current amplitude within *t*_foot_ (Mosharov and Sulzer, 2005)]. HUVEC parameters (mean±s.e.m.) are shown for each in parenthesis (*n* = 762 spikes and 542 pre-spike foot signals from 97 cells). (B) WPB current spikes preceded by pre-spike foot signals. Pre-spike foot signals either increased in a ramp-like fashion (i) showed current fluctuations (ii), or showed a plateau phase (iii). Pre-spike foot signals of large amplitude were also observed, resembling a step-like increase prior to the fast phase of the spike (iv). (Ci,ii) Examples of current spikes lacking pre-spike foot signals. (D) Example of a transient WPB fusion event (Babich et al., 2008). (Di) Top panel shows images of the fusion and morphological transition of a single proregion-EGFP containing WPB, isolated beneath the electrode. Images were acquired at 15 frames/second and correspond to the times indicated by the asterisks. Scale bar: 5 µm. The lower panel shows the time-course for the changes in WPB EGFP fluorescence intensity (grey trace) and current transient (black trace). The current transient is shown on an expanded time scale in Dii.

Fig. 4A shows a typical current spike from a control cell, with each of the measured spike parameters indicated (see also Materials and Methods) and the mean parameter values in parentheses. Pre-spike foot signal parameters are shown in Fig. 4Aii. Current spikes varied in amplitude and shape (Fig. 4B), however, the majority (74.73±1.99%, *n* = 68 cells) showed a clear pre-spike foot signal, often with complex kinetics including ramp-like (Fig. 4Bi), fluctuating (Fig. 4Bii) or more stable plateau-like (Fig. 4Biii) current increases prior to the onset of the fast rise of the main spike. Occasionally, the pre-spike foot signal comprised large amplitude, step-like increases in current (Fig. 4Biv). Fig. 4C shows examples of current spikes lacking pre-spike foot signals. Rarely, small amplitude current increases of variable duration, resembling stand-alone foot signals (Alvarez de Toledo et al., 1993; Chow et al., 1992) were observed (Fig. 4D). In these cases, optical analysis revealed fusion and collapse of the WPB into a spherical structure in which proregion–EGFP was retained (Fig. 4Di, image montage). We next studied the effect of perturbation of total cellular cholesterol on WPB exocytosis, proregion secretion and WPB spike parameters.

### Cholesterol modulates WPB exocytosis and fusion pore dynamics

**Fig. 5.**
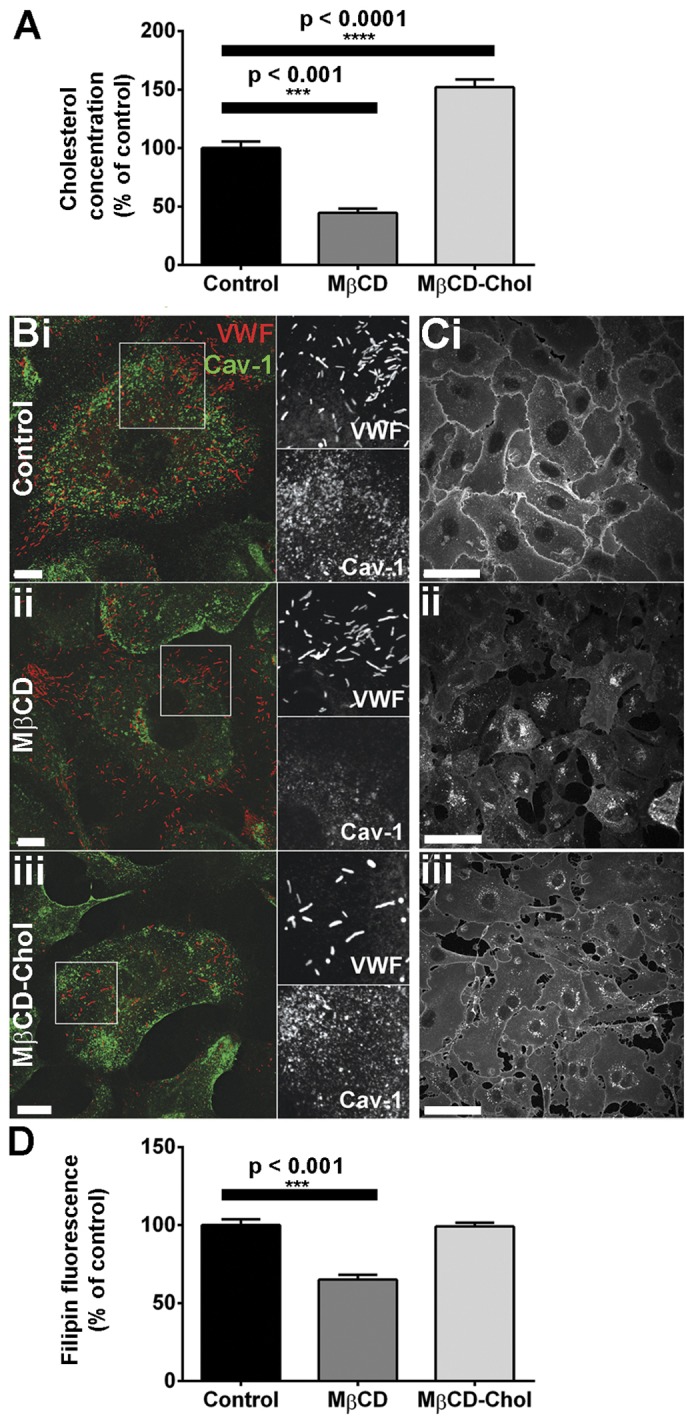
**Depletion or supplementation of HUVEC cholesterol by MβCD.** (A) HUVECs were treated with vehicle (control), 5 mM MβCD or 5 mM MβCD-Chol for 30 minutes and cholesterol content was quantified as described in the Materials and Methods. Data were compared by one-way ANOVA (*n* = 7 experiments for control, *n* = 3 for MβCD and *n* = 12 for MβCD-Chol). (B,C) HUVECs treated as described for A were stained with specific antibodies to VWF (red, sheep Ab) and Cav-1 (green) (B) or filipin (C). (D) Filipin fluorescence was quantified for each treatment condition and was compared by one-way ANOVA (*n* = 75 cells for control, 80 for MβCD and 120 for MβCD-Chol). Scale bars: 10 µm (B), 50 µm (C).

Total cellular cholesterol was reduced or increased by ∼50% by treatment with methyl-β-cyclodextrin (MβCD, 5 mM) or cholesterol-loaded MβCD (MβCD-Chol, 5 mM), respectively (Fig. 5A). To confirm that MβCD treatment depleted plasma membrane cholesterol, we immunolabelled MβCD-treated HUVECs with specific antibodies to the caveolae-resident cholesterol-binding protein caveolin-1 (Cav-1). Cholesterol depletion decreases Cav-1 localisation at the plasma membrane and caveolae flatten (Rothberg et al., 1990). Consistent with this there was a reduction in the punctate pattern of plasma membrane Cav-1 immunoreactivity and an increase in the intensity of perinuclear Cav-1 immunoreactivity (Fig. 5Bi,ii), although WPB numbers and morphology were not visibly altered. In MβCD-Chol-treated cells the pattern of Cav-1 immunoreactivity did not appear substantially altered (Fig. 5Biii). Filipin fluorescence intensity was also significantly reduced in MβCD-treated HUVECs, while in MβCD-Chol-treated cells filipin intensity was similar to controls, although its distribution appeared qualitatively altered with reduced intensity at cell–cell contacts (Fig. 5C,D).

**Fig. 6.**
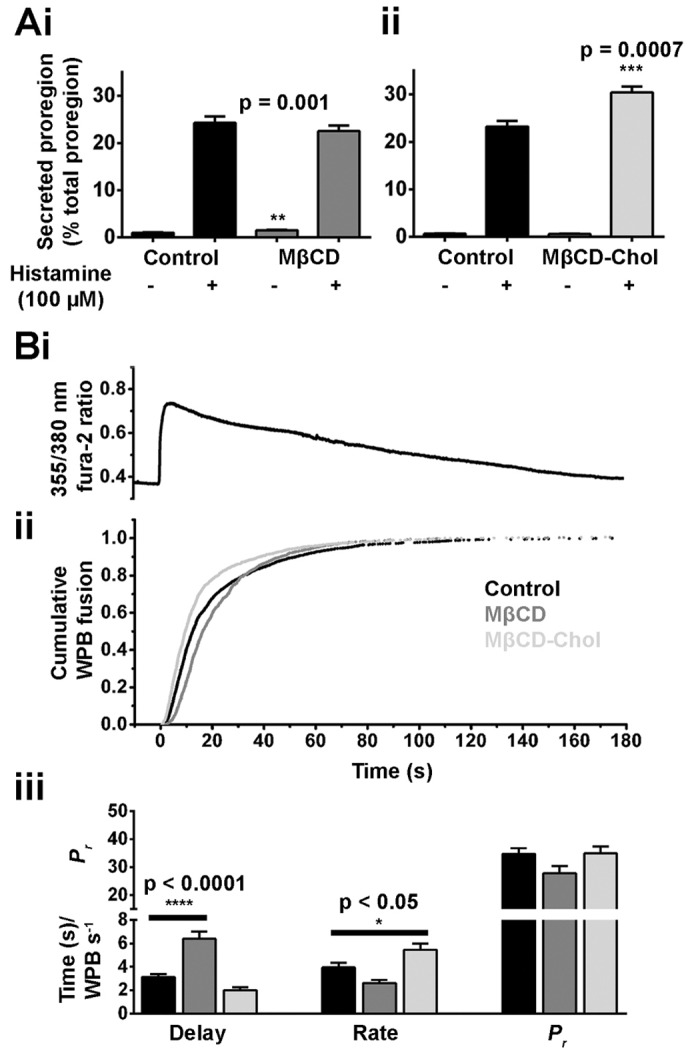
**Perturbation of cellular cholesterol affects hormone-stimulated proregion secretion and WPB exocytosis.** (Ai–ii) Histamine-stimulated secretion of proregion from HUVECs pre-treated for 30 minutes with vehicle (control), 5 mM MβCD or 5 mM MβCD-Chol. Data were compared by Student's *t*-test (*n* = 3 separate experiments for i and ii). (B) Kinetics of histamine-evoked (100 µM) WPB exocytosis following the same treatment as detailed in A. Increases in fura-2 fluorescence ratios were not different between treatment groups; a trace averaged from seven individual cells is shown in Ai. (ii) Cumulative plots of WPB fusion times, normalised to the total number of fusion events for control (black trace, *n* = 2057 events), and cells treated with MβCD (dark grey trace, *n* = 1101 events) and MβCD-Chol (light grey trace, *n* = 2014 events). (iii) Summary of the mean delay between the [Ca^2+^]_i_ rise and first fusion event (seconds), mean maximal rate of exocytosis (WPBs/second) and probability of WPB fusion (*P_r_*, percent, note broken *y*-axis) for control (black bars, *n* = 43 cells), and cells treated with MβCD (dark grey bars, *n* = 35 cells) and MβCD-Chol (light grey bars, *n* = 42 cells). Data were compared by one-way ANOVA.

Histamine-evoked proregion secretion did not change in MβCD-treated cells, but was significantly increased in MβCD-Chol-treated cells (Fig. 6Ai,ii). Optical analysis of individual HUVECs showed that histamine-evoked increases in fura-2 fluorescence ratios were similar in control, MβCD- or MβCD-Chol-treated cells, and an averaged record from seven cells is shown in Fig. 6Bi. However, optical analysis did reveal significant differences in the time course of WPB exocytosis between treatment groups, illustrated in the cumulative plots of exocytotic events in Fig. 6Bii. In MβCD-treated cells there was a significant increase in the delay in the onset of exocytosis although rate and the mean fraction of fluorescent WPBs that underwent exocytosis [a measure of the probability of release, *P_r_* (Bierings et al., 2012)] were not changed (Fig. 6Biii). In MβCD-Chol-treated cells the rate of WPB exocytosis increased significantly (Fig. 6Biii), although delay and *P_r_* were not altered. We next used amperometry to assess the effect of MβCD and MβCD-Chol treatment on the properties of the WPB fusion pore.

**Fig. 7.**
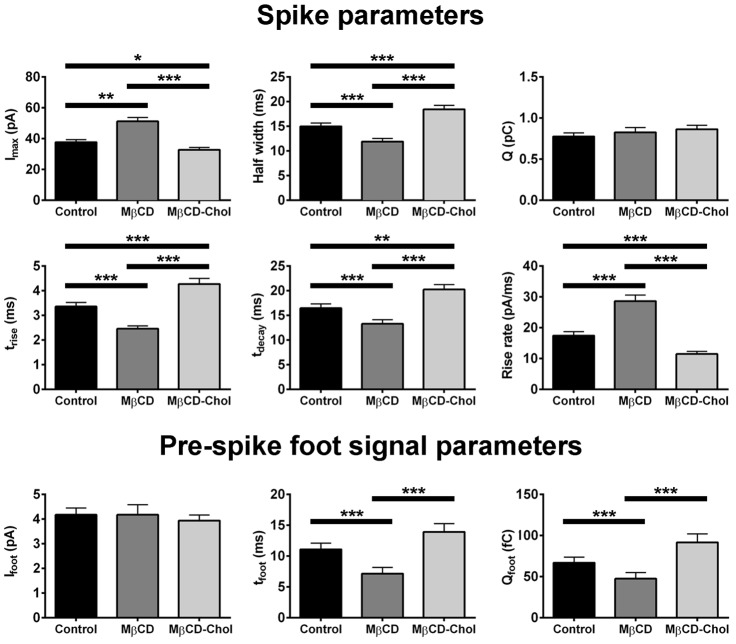
**Cholesterol modulates WPB fusion pore formation and expansion.** Top panels show the mean spike parameters in control (black bars, *n* = 412 spikes, 51 cells), and cells treated with MβCD (dark grey bars, *n* = 405 spikes, 51 cells) and MβCD-Chol (light grey bars, *n* = 401 spikes, 51 cells). Bottom panels show the pre-spike foot signal parameters for control (black, *n* = 303, 51 cells), and cells treated with MβCD (light grey bars, *n* = 303 spikes, 51 cells), and MβCD-Chol (*n* = 301, 51 cells).

WPB spike and pre-spike foot signal parameters recorded from MβCD and MβCD-Chol treated HUVECs are summarised in Fig. 7 (and in supplementary material Table S1C). Cholesterol depletion resulted in a decrease in the spike *t*_1/2_, *t*_rise_ and *t*_decay_ and an increase in the rise rate and *I*_max_, as well as a reduction in the duration of the pre-spike foot signal *t*_foot_. A trend in the opposite direction was seen after cholesterol supplementation. Overall, data indicate that cholesterol depletion increased the rate of fusion pore expansion and destabilised the early fusion pore whereas cholesterol supplementation had broadly the opposite effect.

## Discussion

### WPBs accumulate 5-HT through expression of VMAT1

Electrochemical detection of secretion is dependent on the release of electro-active species during exocytosis (Chow and Von Ruden, 2009), such as monoamines, that are stored within secretory granules of neuronal, neuroendocrine and endocrine cells and blood platelets (Eiden et al., 2004). Accumulation of stored monoamines is driven by the electrogenic transporters VMAT1 and/or VMAT2 on the secretory granule membrane (Eiden et al., 2004). To date, there have been no reports that endothelial cells express a VMAT or that WPBs contain monoamines. Here, we show that HUVECs express VMAT1 and that epitope-tagged VMAT1 localises to WPBs and mediates WPB 5-HT sequestration, as indicated by the abolition of WPB 5-HT immunoreactivity by reserpine (or NH_4_Cl-mediated neutralisation of the acidic intra-WPB environment). An acidic granule lumen is essential for VMAT function, but also promotes the cationic state of 5-HT, which is both less permeable to the granule membrane and is more likely to bind to anionic proteoglycan matrix components (Marszalek et al., 1997). These contribute to the accumulation of secretory granule 5-HT to a high concentration [up to ∼0.5 M (Ge et al., 2009)] and similar processes are likely to occur in WPBs.

The source of the 5-HT in WPBs was the tissue culture fetal calf serum. How might extracellular 5-HT enter HUVECs? Pulmonary and brain endothelial cells express SERT (Brust et al., 2000; Ni and Watts, 2006), however, HUVECs appear to lack a high affinity 5-HT uptake mechanism (Mann et al., 1989) and consistent with this we failed to detect SERT mRNA. However, endothelial cells including HUVECs can express low affinity, high capacity monoamine transporters including PMAT and OCT3 (Barnes et al., 2006; Ferk et al., 2012; Solbach et al., 2011). Although we detected PMAT expression (but surprisingly not OCT3), preliminary experiments using the potent PMAT and OCT3 inhibitor decynium-22 (Engel et al., 2004) failed to abolish WPB 5-HT immunoreactivity, suggesting that 5-HT enters the cell primarily by passive diffusion across the plasma membrane. We failed to detect dopmaine, noradrenaline or adrenaline immunoreactivity in WPBs. These monoamines are present in fetal calf serum, but at much lower levels than 5-HT, equivalent to ∼20 nM for dopamine (Little et al., 2002) and >0.1 nM for noradrenaline or adrenaline (Dibner and Insel, 1981) in full growth medium containing 20% fetal calf serum. The rank order of potency for competitive block of VMAT1-mediated 5-HT transport is 5-HT>dopamine>>noradrenaline, adrenaline (Erickson et al., 1996), hence it is likely that 5-HT is the predominant WPB monoamine under our tissue culture conditions.

The capacity of some cells to sequester 5-HT in WPBs was not unique to HUVECs; we observed the same in human aortic endothelial cells suggesting a possible physiological relevance. Circulating levels of 5-HT are regulated by active 5-HT uptake in platelets (Mercado and Kilic, 2010) and endothelial cells in lung and other organs (Brust et al., 2000; Ni and Watts, 2006). Some endothelial cells (e.g. pulmonary) also make 5-HT (Dempsie and MacLean, 2008), and both receptor-mediated and direct chemical effects of this monoamine are implicated in physiological (e.g. haemostasis) and pathological (e.g pulmonary hypertension) processes within the vasculature (Berger et al., 2009; Dempsie and MacLean, 2008). The tight regulation of the level of circulating 5-HT is therefore essential. Our observation that WPBs sequester and release 5-HT (and potentially other natural or xenobiotic molecules) provides new insight into this regulation. Storage and release of 5-HT by WPBs could contribute to the control of haemostasis and/or might function as part of a protective mechanism to limit cell damage following exposure to high local concentrations of monoamines (e.g. platelet-derived 5-HT at sites of injury).

### Electrochemical analysis of WPB fusion

Current spikes were directly associated with the fusion of individual WPBs. The brief delay between the onset of a current spike and rise in WPB–EGFP fluorescence intensity is probably due to the strong proton buffering of the WPB matrix, estimated at 55 mM pH^−1^ unit (Erent et al., 2007). A striking feature was the high proportion of spikes with a pre-spike foot signal (∼75%), showing that the majority of WPB fusions proceed through an initial narrow fusion pore. For comparison, in other cells the typical frequency of spikes with a pre-spike foot signal is much lower, ranging from 5–12% in mast cells (Alvarez de Toledo et al., 1993), 8–11% in platelets (Ge et al., 2009), and up to 20–35% in chromaffin cells (Schroeder et al., 1996). A factor thought to affect the formation of a restricted fusion pore is secretory granule membrane tension; with a low tension increasing the probability of the formation of a restricted fusion pore (Amatore et al., 2005; Ge et al., 2008). WPBs undergo a profound morphological transition during exocytosis, from a cylindrical shape to a sphere (Erent et al., 2007). A sphere has a smaller surface area than a cylinder of the same volume, and so it is conceivable that for a brief period the WPB membrane tension decreases, favouring the formation of an initially restricted pore. This morphological transition, unique to WPBs, might therefore account for the unusually high proportion of pre-spike foot signals.

Infrequently, we observed current changes reminiscent of stand-alone foot signals, thought to represent the failure of a restricted fusion pore to expand (Fig. 4C), concomitant with WPB fluorescence intensity changes that indicated lingering-kiss fusion (Babich et al., 2008). WPB lingering-kiss fusion was first identified optically and is characterised by the formation of a restricted fusion pore of ∼12 nm that does not expand, thereby preventing the release of fluorescent cargo of high molecular mass (Babich et al., 2008). Such events represent ∼10% of WPB fusions and are readily detected optically, where most or all fluorescent WPBs can be observed simultaneously. In contrast, the electrode samples only a tiny area of the upper surface of the endothelial cell, reducing the chances of detecting these events. Consequently the characterisation and interpretation of putative stand-alone foot signals was difficult. One feature of such events was a relatively large *I*_max_, e.g. 37.31 pA for the example in Fig. 4C, a value within the range of spike amplitudes for full fusion. This probably reflects the large size of the lingering-kiss fusion pore [∼12 nm (Babich et al., 2008)], which might not retard 5-HT release substantially. Indeed, a large but not fully expanded pore might account for the unusually large amplitude pre-spike foot signals that we also observed (e.g. Fig. 4Biv). Together, the data indicate that in most cases WPB fusion proceeds through an initial restricted fusion pore, which might ‘flicker’ reversibly before closing or fully expanding.

### Cholesterol modulation of WPB exocytosis

Plasma membrane cholesterol is involved in multiple aspects of exocytosis including organisation of t-SNARE machinery (Churchward and Coorssen, 2009) and modulation of membrane curvature during fusion pore formation (Churchward et al., 2008). Cholesterol depletion inhibits exocytosis in a range of cell types, an effect attributed to the disruption of t-SNARE proteins (Churchward and Coorssen, 2009). We observed a highly significant increase in the delay in the onset of WPB exocytosis (Fig. 6Biii), suggesting that cholesterol depletion might also disrupt t-SNARE proteins in endothelial cells. However, the mean extent of WPB exocytosis (*P_r_*) and proregion secretion was not affected by cholesterol depletion, consistent with a previous study assaying extracellular VWF string formation (Huang et al., 2010). Taken together with reports highlighting the difficulty in blocking VWF secretion by depletion of t-SNARE proteins (Pulido et al., 2011), these results suggest that WPB exocytosis is remarkably resilient to factors that may disrupt plasma membrane composition.

Following cholesterol supplementation, we observed an increase in the rate of WPB exocytosis and the extent of proregion secretion, however, paradoxically not in *P_r_* (although this parameter became more variable). This difference is likely to reflect a small sample size for optical data, compared to biochemical analysis, which assays secretion from millions of cells. These results might also reflect changes in the localisation of t-SNARE proteins; because cholesterol has been implicated in the organisation of fusion sites (Churchward and Coorssen, 2009), supplementation of cholesterol might increase the concentration of t-SNARE proteins allowing increased access of WPBs to plasma membrane fusion machinery.

Cholesterol is also thought to be directly involved in the modulation of the exocytotic fusion pore by stabilising intermediate structures (including the early restricted fusion pore) during bilayer fusion through its ability to promote negative membrane curvature (Chen and Rand, 1997; Churchward and Coorssen, 2009). In agreement with data from platelets, PC12 and chromaffin cells (Ge et al., 2010; Koseoglu et al., 2011; Wang et al., 2010; Zhang et al., 2009), we observed a decrease in pre-spike foot duration following cholesterol depletion, providing further evidence for the role of cholesterol in the formation and stabilisation of the restricted fusion pore. In addition, we observed an increase in the rate of fusion pore expansion, in agreement with data from platelets (Ge et al., 2010) but in contrast to data from PC12 or chromaffin cells (Wang et al., 2010; Zhang et al., 2009). The basis for the discrepancies between different cell types remains unclear.

Our results demonstrate that cholesterol plays multiple roles in WPB exocytosis, influencing the overall extent of exocytosis, and both fusion pore formation and expansion. It is not clear whether acute changes in circulating cholesterol levels substantially impact endothelial cell cholesterol, and one might speculate that endothelial cells are resilient to sudden changes. However, the effects seen here for hormone-evoked WPB exocytosis could reflect an underlying mechanism to account for increased circulating VWF observed together with chronically elevated plasma cholesterol levels (Blann et al., 1995; Pérez-Jiménez et al., 1999) and which contribute to a higher risk of vascular disease (Jansson et al., 1991; Lip and Blann, 1997).

## Materials and Methods

### Tissue culture, transfections, ELISAs, immunocytochemistry, antibodies, DNA constructs, RT-PCR and reagents

HUVECs were obtained, cultured in full growth medium and transfected as previously described (Bierings et al., 2012). Dialysed medium comprised full growth medium with 20% dialysed fetal calf serum (24 hours, 4°C, 0.15 M NaCl, 10,000 MWCO SnakeSkin tubing; Thermo Fisher Scientific, Cramlington, UK). Serum-free growth medium was supplemented with 20 mM HEPES (pH 7.4) and 2% BSA. Immunocytochemistry and ELISAs for the proregion were performed as previously described (Bierings et al., 2012). Sources and dilutions of all antibodies used are in supplementary material Table S1A. Proregion–mEGFP and P-selectin–mCherry were made as previously described (Bierings et al., 2012 and references therein), EGFP–VMAT1 (Essand et al., 2005) was provided by Professor Giandomenico (University of Uppsala, Sweden). mCherry–VMAT1 was made by exchanging EGFP with mCherry as an *Nhe*I/*Bsr*GI fragment cut from pmCherry-C2 (Clontech, Mountain View, CA) and cloned into *Nhe*I/*Bsr*GI digested EGFP–VMAT1. EGFP–VAChT was from Professor Takahashi (Department of Biochemistry, Kitasato-University School of Medicine, Japan). RNA was extracted from HUVECs, BON cells or human adrenal tissue (from Medical Solutions Plc, Nottingham, UK, used in accordance with the terms of the MRC NIMR Human Tissue Authority licence) as previously described (Bierings et al., 2012). RT-PCR was performed using the SuperScript III Reverse Transcriptase kit (Invitrogen, Grand Island, NY, USA). Primer sequences for PMAT, VMAT1, VMAT2, SERT and OCT3 are in supplementary material Table S1B. Fura-2 AM was from Molecular Probes (Eugene, OR, USA). Other reagents were from Sigma-Aldrich (Gillingham, UK) unless otherwise stated.

### Determining the source of WPB-associated 5-HT

Non-transfected or EGFP–VMAT1-expressing HUVECs (16 or 48 hours post-transfection) were exposed to vehicle or 10 mM NH_4_Cl for 5 minutes in serum-free growth medium, followed by incubation in dialysed medium or serum-free growth medium for 5 to 16 hours in the absence or presence of 5-HT (0.03–3 µM) prior to immunocytochemistry.

### Cholesterol depletion and supplementation

Non-transfected, proregion–EGFP-, or proregion–EGFP- and mCherry–VMAT1-expressing HUVECs (48 hours post-transfection) were exposed to vehicle (distilled H_2_O), 5 mM MβCD or 5 mM MβCD-Chol in serum-free growth medium for 30 minutes. MβCD-Chol was made as previously described (Klein et al., 1995). Filipin (10 µg/ml) staining was performed on fixed cells as for immunocytochemistry (Bierings et al., 2012). Total cellular cholesterol was quantified using an Amplex Red cholesterol assay kit according to the manufacturer's instructions (Invitrogen, Grand Island, NY, USA) using a micro-plate reader (Molecular Devices Corporation, Sunnyvale, CA, USA).

### Live-cell fluorescence imaging and intra-WPB pH measurements

Imaging of fura-2, proregion–EGFP-containing WPBs and measurements of intra-WPB pH were carried out using both total internal reflection fluorescence (TIRF) and epifluorescence microscopy as described previously (Erent et al., 2007). The mean fraction of fluorescent WPBs that underwent exocytosis was taken as a measure of the probability of release, *P_r_*, as previously described (Bierings et al., 2012). Images were acquired at 30–100 frames/second, image capture was synchronised with illumination using Winfluor software (http://spider.science.strath.ac.uk).

### Carbon fibre microelectrode fabrication, preparation and amperometric recording

Electrodes were fabricated as described in Pike et al. (Pike et al., 2009). Electrodes were sealed with epoxy resin (∼90°C), cured overnight at room temperature and then baked at 100°C for 2 hours and 150°C for ∼24 hours. This was repeated but with an extended curing period of 2–5 days at 150°C. Electrode tips were bevelled to 45° on a microelectrode beveller with a coarse diamond abrasive plate (Sutter Instruments, Novato, CA) and soaked in 2-propanol for ∼20 minutes. Electrodes were tested as previously described (Schulte and Chow, 1996) and those with a stable baseline current used. Electrodes were re-bevelled after 1–3 experiments. Electrodes were backfilled with 150 mM KCl solution and connected to an Axopatch 200B amplifier (Axon Instruments, Foster City, CA, USA) in voltage clamp configuration, and held at +700 mV versus a silver/silver chloride reference electrode, for detection of 5-HT, dopamine, noradrenaline and adrenaline. Data were acquired at 10 KHz, filtered at 5 KHz, and recorded using Winfluor software. Prior to amperometry, cells co-expressing mCherry–VMAT1 and proregion–EGFP (48 hours post transfection) were exposed to 100 µM 5-HT in serum-free growth medium, to enhance WPB-5-HT loading. Cells were fura-2-loaded as previously described (Erent et al., 2007). Stimulation was by gentle mechanical pressure of the electrode onto the cell. Fura-2 355 nm fluorescence was monitored to determine if stimulation caused cell rupture (indicated by a quench in 355 nm fluorescence), however this was not seen to occur.

### Data analysis

Amperometric data were analysed using a custom written macro for Igor Pro (Mosharov and Sulzer, 2005) (http://www.sulzerlab.org/download.html). Traces were low-pass filtered (1000 Hz Gaussian filter) followed by binomial smoothing (500 Hz). Thresholds for spike and foot detection were five and two times the root-mean-square of the background current, respectively. Spike parameters determined included: *I*_max_, maximum spike amplitude; *Q*, integrated spike charge; *t*_1/2_, spike width at half the spike maximum; *t*_rise_, 25–75% rise time of the spike; rise slope, rate of the rising phase of the spike; and *t*_decay_, duration of the decay phase of the spike between 75 and 25% of the amplitude. Pre-spike foot signal parameters were: *I*_foot_, average amplitude; *t*_foot_, duration and *Q*_foot_, integrated charge. Spikes with a *t*_rise_ greater than 3 s.d. + mean t_rise_ were rejected, as previously described (Mosharov and Sulzer, 2005). Statistical analysis and comparison of log-transformed current spike parameters was carried out by one-way ANOVA, with a Bonferroni post test to compare pairs of data sets, and for secretion data by Student's *t*-test or one-way ANOVA as indicated, in GraphPad Prism Version 5 (GraphPad Software Inc., La Jolla, USA). Image analysis was carried out in Winfluor software or using custom-written plugins and macros in ImageJ software (http://rsb.info.nih.gov/ij/) as previously described (Bierings et al., 2012; Erent et al., 2007). Data were plotted in Microcal Origin 8 (OriginLab Corp., Northampton, MA, USA). Data are expressed as mean±s.e.m.

## Supplementary Material

Supplementary Material
