# Supplementary material for: Characterisation of Weibel–Palade body fusion by amperometry in endothelial cells reveals fusion pore dynamics and the effect of cholesterol on exocytosis
Source: J Cell Sci. 2013 Dec 1;126(23):5490–9. doi: 10.1242/jcs.138438 (PMC3843139; doi:10.1242/jcs.138438)
Supplement: Supplementary Material [file supp_126_23_5490__index.html]

Characterisation of Weibel–Palade body fusion by amperometry in endothelial cells reveals fusion pore dynamics and the effect of cholesterol on exocytosis — Supplementary Material 

# Characterisation of Weibel–Palade body fusion by amperometry in endothelial cells reveals fusion pore dynamics and the effect of cholesterol on exocytosis

## JCS138438 Supplementary Material

**Files in this Data Supplement:**

- **Supplementary Material PDF**
