# Supplementary material for: Characterisation of Weibel–Palade body fusion by amperometry in endothelial cells reveals fusion pore dynamics and the effect of cholesterol on exocytosis
Source: J Cell Sci. 2013 Dec 1;126(23):5490–9. doi: 10.1242/jcs.138438 (PMC3843139; doi:10.1242/jcs.138438)
Supplement: Supplementary Material [file supp_126.23.5490_JCS138438.pdf]

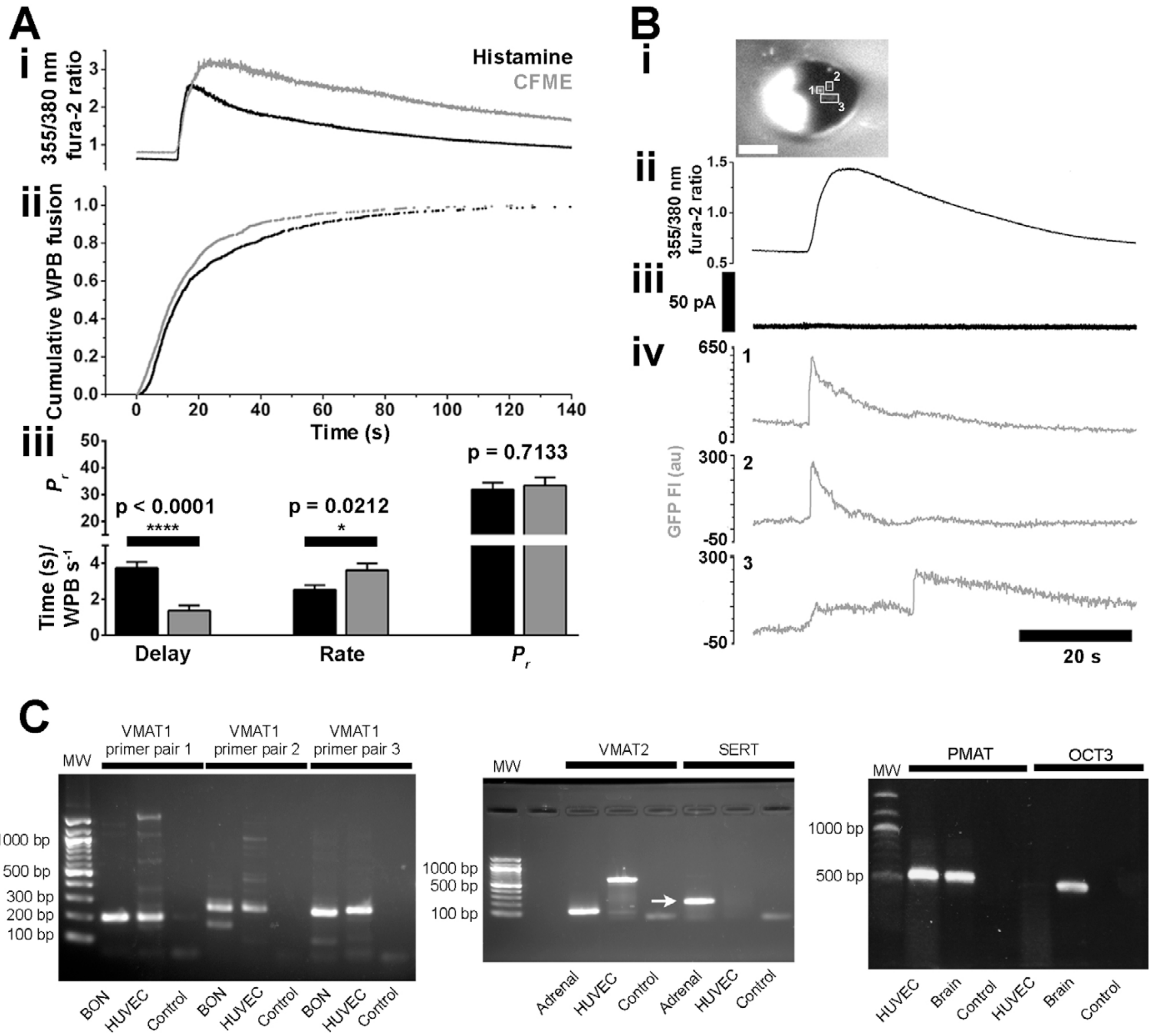

Fig. S1. A: *Mechanical stimulation of HUVEC with a carbon fibre microelectrode induces WPB exocytosis.* The response of endothelial cells to mechanical stimulation has been extensively studied, with mechanical pressure, changes in shear stress or endothelial cell stretching inducing increases in $[Ca^{2+}]_i$ (Demer et al., 1993; Diamond et al., 1994; Goligorsky, 1988; Sigurdson et al., 1993). The mechanisms underlying the Ca^{2+} increase are thought to involve both activation of cell surface cation cannels and release of intracellular Ca^{2+} (Mendoza et al., 2010; Nilius et al., 2003). Despite these findings, including the observation that mechanical stretch induces VWF release (Galbusera et al., 1997), a direct analysis of WPB exocytosis following mechanical stimulation has not been reported. (Ai) shows a representative 355/380nm fura-2 fluorescence record in single proregion-EGFP expressing HUVEC during mechanical (grey trace) or histamine (100 µM, black trace) stimulation. (Aii) Cumulative plots of WPB fusion times, normalised by the total number, for mechanical (grey traces, 1087 events, 20 cells) or histamine (black traces, 836 events, 26 cells) stimulation. (Aiii) mean (± SEM) delay between the $[Ca^{2+}]_i$ rise and the first fusion event (s); mean maximal rate of exocytosis (WPBs s^{-1}) and the probability of WPB exocytosis (P_r, percent, note broken y axis) for histamine (black bars, n = 26 cells) and mechanical (grey bars, n = 20 cells) stimulation. Together the data show that mechanical stimulation results in a rapid release of WPBs with a probability of release (P_r) (see (Bierings et al., 2012)), similar to that obtained for a maximal concentration of the physiological stimulus histamine. **B:** *WPB exocytosis is not associated with current spikes***.** (Bi) Image of an electrode positioned over a region of a HUVEC containing multiple fluorescent WPBs (fluorescence intensity changes due to fusion for the three WPBs indicated are plotted in (Biv)). Scale bar is 5 µm. (Bii) Fura-2 ratio increase following mechanical stimulation. (Biii) Electrode current recorded at +700 mV. (Biv) WPB fluorescence intensity of the three WPBs indicated in (Bi). Fluorescence intensity increases are due to fusion of the WPBs with the plasma membrane. **C:** *RT-PCR analysis reveals VMAT1 expression in HUVEC***.** (Left) Three sets of diagnostic primers to VMAT1 were tested on HUVEC RNA, with BON cell RNA as a positive control. The identity of the PCR products amplified with VMAT1 primers was confirmed by sequencing. (Middle) Primers to VMAT2 and SERT were tested on HUVEC RNA and human adrenal RNA, no band of the correct size was observed for either SERT or VMAT2. (Right) Diagnostic primers to PMAT and OCT3 were tested on HUVEC RNA, with brain RNA as a positive control. No band was observed for OCT3 in HUVEC RNA. Identity of the PCR product amplified with PMAT primers was confirmed by sequencing. Lanes marked control contained no template RNA.

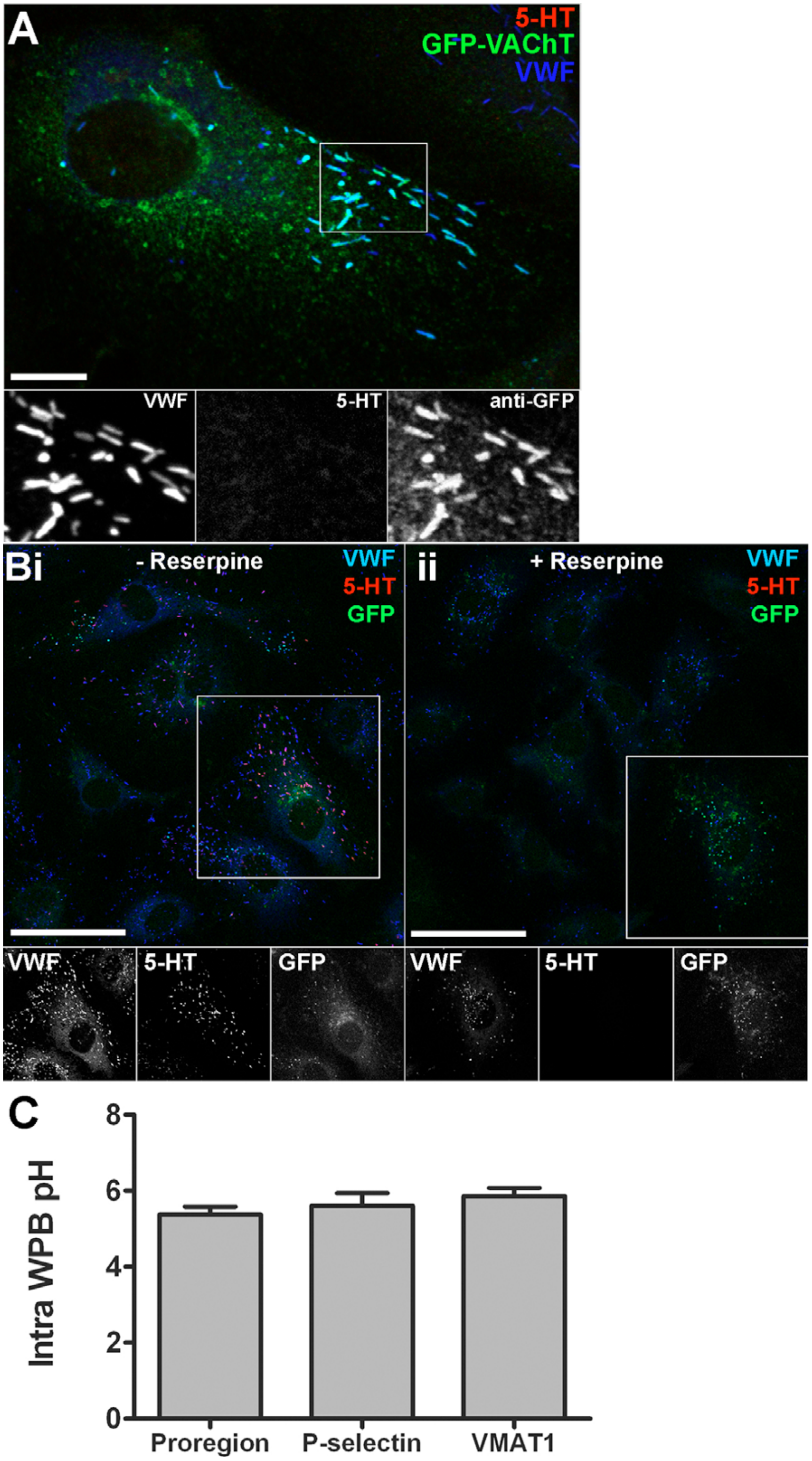

Fig.S2. A: *EGFP-VAChT localises to WPBs but does not result in WPB-5-HT-IR.* Images show a HUVEC 48 hours post-transfection with EGFP-VAChT, fixed and immunolabelled for endogenous VWF (blue, rabbit Ab), 5-HT (red) and GFP (green). Scale bar is 10 µm. Gray scale images for each channel, taken from the region indicated by white box, are shown below on an expanded scale. **B:** *Reserpine abolishes WPB-5-HT-IR in EGFP-VMAT1 expressing HUVEC.* EGFP-VMAT1 expressing HUVEC (48 hours post-transfection) were treated with (Bi) or without (Bii) 100 µM reserpine for 60 minutes in full growth media. Cells were fixed and immunolabelled for VWF (blue, rabbit Ab), 5-HT (red) and EGFP (green). Gray scale images for each channel, taken from the region indicated by the white box, are shown below. Scale bars are 50 µm. **C:** *Expression of mCherry-VMAT1 does not alter intra-WPB pH***.** HUVEC were nucleofected with proregion-mEGFP (Proregion), proregion-mEGFP and P-selectin-mCherry (P-selectin, as a control for the double transfection) or proregion-mEGFP and mCherry-VMAT1 (VMAT1) and the intra-WPB pH determined by the NH_4Cl pulse technique using intra-WPB EGFP (fused to proregion) as the WPB-specific pH indicator, as previously described (Erent et al., 2007). The pH of 20 WPBs was calculated from four cells for each condition. Data expressed as mean ± SD.

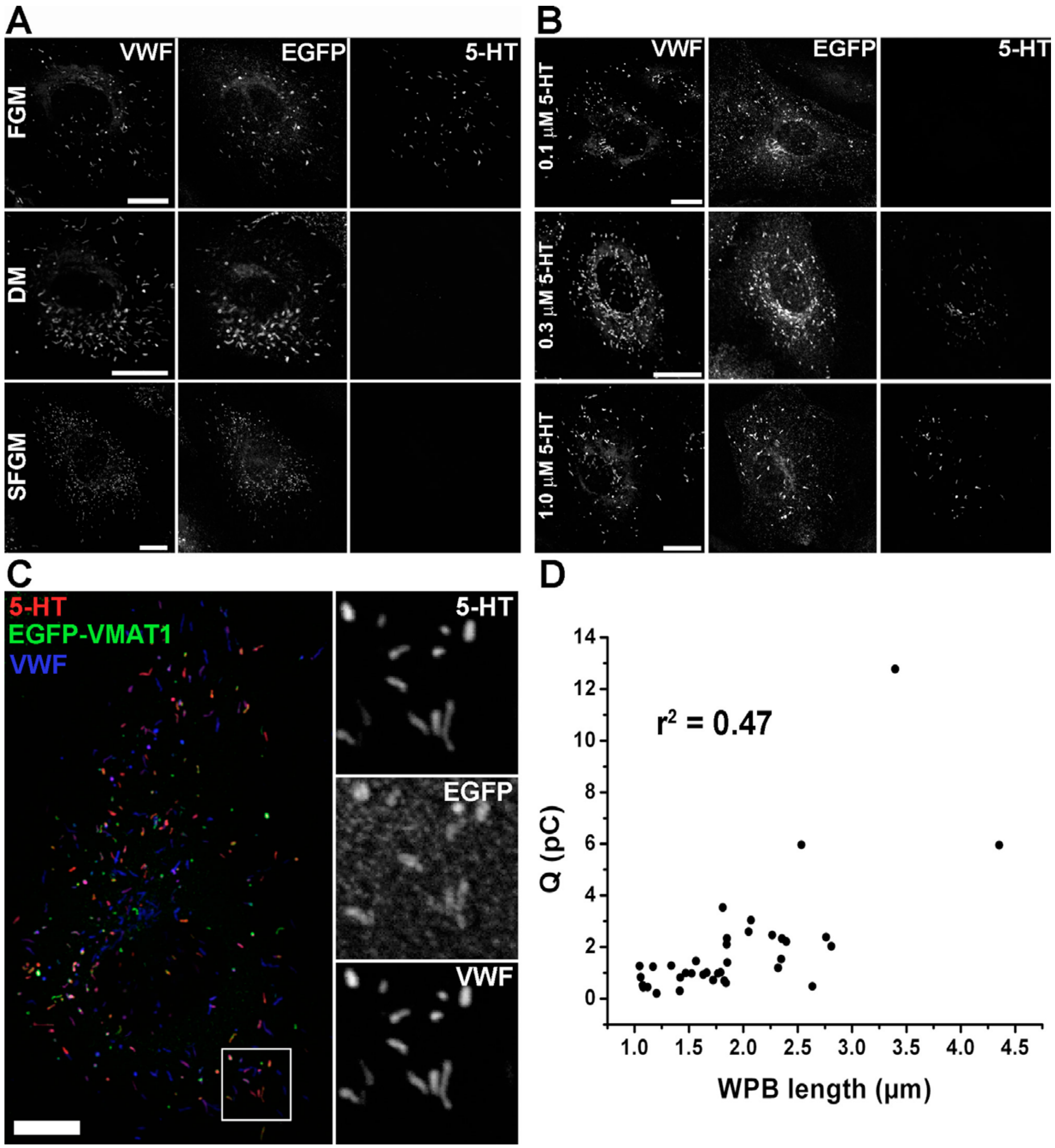

Fig. S3. A: *Foetal calf serum is the source for WPB-5-HT-IR in EGFP-VMAT1 expressing HUVEC***.** Images show EGFP-VMAT1 expressing HUVEC (16 hours post-transfection) immunolabelled for VWF (left, rabbit Ab), GFP (middle) and 5-HT (right). Cells were exposed to 10 mM NH_4Cl for 30 minutes to discharge endogenous 5-HT-IR from WPBs, then incubated in full growth media (FGM), dialysed media (DM) or serum free growth media (SFGM) (see Materials & Methods) for 5 hours prior to fixation. Scale bars are 10 µm. **B:** *Supplementing dialysed media with low concentrations of 5-HT restores WPB-5-HT-IR in EGFP-VMAT1 expressing HUVEC.* Images show EGFP-VMAT1 expressing HUVEC (16 hours post-transfection) immunolabelled for VWF (left, rabbit Ab), GFP (middle) and 5-HT (right). Cells were exposed to 10 mM NH_4Cl for 30 minutes to discharge endogenous 5-HT-IR from WPBs, then incubated for 5 hours in DM supplemented with 5-HT at the concentrations indicated, prior to fixation. Scale bars are 10 µm. **C:** *WPB-5-HT-IR is retained for extended periods in EGFP-VMAT1 expressing HUVEC.* EGFP-VMAT1 expressing HUVEC (16 hours post-transfection) immunolabelled for VWF (blue, rabbit Ab), GFP (green) and 5-HT (red). Cells were exposed to 10 mM NH_4Cl for 30 minutes to discharge endogenous 5-HT-IR from WPBs, then incubated for 5 hours in dialysed media supplemented with 1.0µM 5-HT, followed by a further 16 hours in dialysed media prior to fixation. Scale bar is 10 µm. Gray scale images for each channel, taken from the region indicated by white box, are shown below on an expanded scale. **D:** *WPB spike charge (Q) is correlated with WPB length***.** An important consideration during analysis of current spikes is whether the concentration of oxidisable molecules is constant within all granules in the population (Finnegan et al., 1996). To determine if this was the case for WPBs, we assessed whether WPB volume and current spike quantal size (Q) were correlated. The main determinant of WPB volume is the organelles length. The figure shows a scatter plot of Q against WPB length (n = 38), taken from imaging and amperometry experiments in which individual WPBs could be clearly visualised, allowing their lengths to be measured and plotted against the resulting spike Q recorded following fusion. We found that WPB length was significantly correlated with Q ($p < 0.0001$, r2 = 0.47), indicating that the concentration of oxidisable molecules within WPBs is essentially constant irrespective of WPB size.

Table S1

A: Antibodies

	Species	Manufacturer	Clone / catalogue number	Dilutions used for IF
5-HT	Mouse mAb	Abcam	ab16007	1:50
Cav-1	Rabbit pAb	BD Transduction Laboratories	610059/60	1:200
VWF	Rabbit pAb	DAKO	A0082	1:10000
VWF	Sheep pAb	Serotec	AHP062	1:10000
Proregion	Mouse mAb	Previously described	See (Hewlett et al., 2011)	N/A
Proregion	Rabbit pAb	Previously described	See (Hewlett et al., 2011)	N/A
GFP	Sheep pAb	Biogenesis	4745-1051	1:250

B: RT-PCR primers

**Target**	**Primer**	**Sequence**	**Amplicon (bp)**
**VMAT1**	EC10-f	5’- gtggtggtattcgtcgcttt -3’	199
	EC11-r	5’- ccacggtgttgttgttgaag -3’	
**VMAT1**	EC12-f	5’- cttcaaaggctgtgatgcaa -3’	253
	EC13-r	5’- gctcgtcctctctcatggtc -3’	
**VMAT1**	EC14-f	5’- atgggagtgtctacgccatc -3’	244
	EC15-r	5’- ggcttctgggttgcatacat -3’	
**VMAT2**	EC16]-f	5’- agaccatgtgttcccgaaag -3’	126
	EC17-r	5’- gaagagcacaaagccacctc -3’	
**SERT**	EC24-f	5’- caccctagtggctgagcttc -3’	214
	EC25-r	5’- cagtgcgagctccatgtaaa -3’	
**PMAT**	PMAT-fwd2	5’- cgccatctactttgcgatgc -3’	552
	PMAT-rev2	5’- cactaacaggtgcagcagga -3’	
**OTC3**	OTC3-fwd3	5’- taaccattgagcgccttgga -3’	472
	OTC3-rev3	5’- aaggtgagagcgggaaactg -3’	

C: Current spike and pre-spike foot signal parameters recorded from control and cholesterol depleted or supplemented HUVEC

**Spike parameters**	**Control**<br>**n = 412**	**+ 5 mM MβCD**<br>**n = 405**	**+ 5 mM MβCD-Chol**<br>**n = 401**
**I_{max} (pA)***	37.67 ± 1.64	51.27 ± 2.42	32.78 ± 1.45
**$t_{1/2}$ (ms)***	14.98 ± 0.70	11.90 ± 0.64	18.45 ± 0.83
**Q (pC)**	0.77 ± 0.04	0.82 ± 0.06	0.86 ± 0.05
**t_{rise} (25-75%) (ms)***	3.36 ± 0.16	2.46 ± 0.11	4.27 ± 0.23
**t_{decay} (25-75%) (ms)***	16.47 ± 0.87	13.31 ± 0.81	20.27 ± 1.01
**Rise rate (pA/ms)***	17.45 ± 1.29	28.66 ± 1.90	11.48 ± 0.84

**Pre-spike foot signal parameters**	n = 303	n = 303	n = 301
**I_{foot} (pA)**	4.18 ± 0.27	4.18 ± 0.41	3.94 ± 0.23
**t_{foot} (ms)***	11.09 ± 17.46	6.55 ± 0.83	13.93 ± 1.34
**Q_{foot} (fC)***	68.29 ± 6.92	47.60 ± 7.48	91.67 ± 10.24

Data are expressed as mean ± SEM. * indicates parameters that are significantly different between treatment groups as analysed by one-way ANOVA.
